# Supplementary material for: Exploring factors influencing nurses’ attitudes towards their role in dental care
Source: PLoS One. 2023 Jul 20;18(7):e0288927. doi: 10.1371/journal.pone.0288927 (PMC10358942; doi:10.1371/journal.pone.0288927)
Supplement: Attachment — Submitted filename: Response to Reviewers.docx [file pone.0288927.s002.docx]

We thank the Reviewers for their time and their valuable comments. Please find below our responses one by one to all comments and concerns raised.

| Editor | Comments | Authors’ response |
| --- | --- | --- |
|  | A rebuttal letter that responds to each point raised by the academic editor and reviewer(s). You should upload this letter as a separate file labeled 'Response to Reviewers'.  A marked-up copy of your manuscript that highlights changes made to the original version. You should upload this as a separate file labeled 'Revised Manuscript with Track Changes'.  An unmarked version of your revised paper without tracked changes. You should upload this as a separate file labeled 'Manuscript'. | As requested here are our responses to all comments raised one by one.  We also uploaded a revised document with track changes and highlighted text in addition to the manuscript. |
|  | 1. Please ensure that your manuscript meets PLOS ONE's style requirements, including those for file naming. | We followed the journal guidelines. |
|  | 2. Please update your submission to use the PLOS LaTeX template. | We had a problem with the latex template, we contacted the [latex@plos.org](mailto:latex@plos.org) and the editorial office requesting help, but no response. However you followed the instructions for authors. |
|  | 3. Please amend your current ethics statement to address the following concerns:  a) Did participants provide their written or verbal informed consent to participate in this study?  b) If consent was verbal, please explain i) why written consent was not obtained, ii) how you documented participant consent, and iii) whether the ethics committees/IRB approved this consent procedure | We have now updated the ethics statement as requested.  a) in this online survey, all voluntary participants gave their consent electronically before proceeding to the questionnaire  b) consent was electronic/written  c) the procedure was approved by the Ethical Committee |
|  | 3. We note that you have indicated that data from this study are available upon request. PLOS only allows data to be available upon request if there are legal or ethical restrictions on sharing data publicly. | The survey included an open comment box where participants provided their personal experience (barriers and facilitators) in provision of dental care within their institutions. This data will be used in a future qualitative study. Sharing such data might be unacceptable by the referred facilities/ hospitals.  In addition, we did not inform the participants that their responses will be publicly available, causing ethical restriction. |
|  | In your revised cover letter, please address the following prompts:  a) If there are ethical or legal restrictions on sharing a de-identified data set, please explain them in detail (e.g., data contain potentially sensitive information, data are owned by a third-party organization, etc.) and who has imposed them (e.g., an ethics committee). Please also provide contact information for a data access committee, ethics committee, or other institutional body to which data requests may be sent.  b) If there are no restrictions, please upload the minimal anonymized data set necessary to replicate your study findings as either Supporting Information files or to a stable, public repository and provide us with the relevant URLs, DOIs, or accession numbers. | We added the ethical restrictions regarding sharing the data to the cover letter. |

| **#** | **Comments by Reviewer** | **Authors’ responses** |
| --- | --- | --- |
| Reviewer 1 | The topic is interesting and probably will show good findings if it was designed with an adequate method. The following comments are reported in the review of this article. | We thank the Reviewer for the positive feedback and valuable comments. |
|  | Page 3, background, first paragraph: I expect to find some text discussing the objective of the study which is the attitude of the nurses BUT not related to dental caries. This should be totally changed to cope with the aim of the study rather than the nature of dental caries. | We thank the Reviewer for the valuable suggestion. We have amended the introduction and elaborated more on nurses’ attitudes. |
|  | Page 4, Background, line 90: This sentence is not of clear expression. | We have now amended the sentence to clarify it. |
|  | Page 4, Background lines 91-92: these sentences did not show any correlation with the previous text. | We have amended the sentences. |
|  | Page 4, Background line 106: The aim of the study was different from what was stated in the abstract, therefore, should be corrected and unified. The one in the abstract probably sounds better. | We thank the Reviewer for the comment. We have now corrected and unified the aim in line with the abstract. |
|  | Generally saying: the introduction was not properly written and shows incoherence between sentences and ideas. | We have now amended the introduction completely as suggested by the Reviewer. |
|  | Page 5, Method, sample size calculation, line 127: if the Sample size was calculated to be 377 nurses, based on an assumption of 50 percent of nurses, So what methods were used to inflate the sample to 525 as mentioned in the abstract?  This should be clearly described. | We used a snowball sampling technique; the distribution/sharing of the online survey was done spontaneously by multiple respondents at the same time. As such we did not have estimates on responses as for those of face to face or one to one recruitment. We kept the survey link active for one month and all responses were then considered.  We added this clarification to the method section (Page 6 lines 137-140). |
|  | Page 5, Methods, data collection tool, line 135:  How the investigators assured that the respondents were nurses? and how was the process conducted to reach a nurse and not another person? | Through a short-listed personal connection (collected from hospital websites) we reached out for nurses who were requested to share the survey link with registered nurses practicing in Eastern Saudi Arabia only. Any responses from other health professionals were excluded. |
|  | Page 5, methods, line 138: “while the study’s outcome was based on their attitudes.”  this outcome is vague. When do we say attitude what? should be rephrased clearly. | We thank the Reviewer for catching this, the sentence was incomplete and is now rephrased (Page 6 lines 141&142). |
|  | Page 6, Methods, Assessment of attitude, line 157: The calculation was incorrect based on what was stated in the description. If using 5 scales Likert score for 14 questions with a 0 score for strongly disagree, the numbers will be 0, 1, 2, 3, and 4, and no number 5. So, the total score will be 56 and not 70 as mentioned in their description. This will totally change the calculation of the attitude level and basically will move all the results that need to be re-calculated in the study. | We thank the Reviewer for catching this. We did use a 5-point Likert scale with score of five for strongly agreeing and score one for strongly disagreeing; there was a mistake in the previous method part (a score from 0 to 4). All results presented are based on the scores from 1 to 5.  We have now corrected the method section (Page 7 line 170). |
|  | Page 7, Results, table 1: How the investigator approached those non-Saudi nurses, as they used the same version of the questionnaire or a different language? | The survey was administered in both Arabic and English languages. Each question was written in Arabic and English.  We added this to the methodology (Page 6 lines 137-139). |
|  | Page 8, line 181:  Results, Figure 1 is demonstrating the source of oral health information which was not mentioned in the methods. and figure 2, the data was not clear. | We thank the Reviewer for catching this. We added the sources of oral health knowledge (figure 1) as a part of background information (Page 6 lines 151-154).  Figure 2 is now presented as a bar chart. |
|  | Page 9, results, line 202: The total inserted in table 3 is the repetition of the same result in table 1. Therefore, it is better to stick to one of the findings and omit the other. | Table 1 has now been removed to avoid repetition. |
|  | Page 10, Results, line 221: based on the wrong calculation of the total score and the categorical score of the attitude, the rest of the findings will be inadequate. Therefore, it needs to repeat the cut-off of goodness for the attitude and then again make your analysis on the attitude towards oral health section. | As previously explained this was a typographic error. The results are based on the correct scoring from 1 to 5. |
|  | Discussion, page 13, line 241: "Saudi nurses showed overall positive attitudes towards their role in dental care, and the majority were willing to provide oral health education"This fact should be revised after the correction of the calculation of the cut-off point and the scores used in this part. | The cut-off point is correct, as mentioned in response to the Reviewer’s previous comment. The typographic mistake has now been corrected (Page 7 line 170). |
|  | Discussion, page 13, line 255: as the author stated that the positive role of the nurses is comparable with other studies is good but also needs to show the consistency or differences with other studies by percentage at least for each study. | Percentages were added for the studies from Finland and Ethiopia. Since the study from Australia did not report actual percentage it was replaced by a study from Korea (*Lee YJ, et al.(2019).* |
|  | Limitation, page 16: among the limitations that were found in the study is the use of online distribution of the questionnaire for a specific profession without control whether they are students in the dental college or other general population or even from outside the country. This part showed an inflated sample size without any clarification of the reason except it is a snowball method. | We thank the Reviewer for the comment. We have clarified in the method section according to your previous comments that the study included only nurses. The inflated sample was mainly due to the nature of the sampling technique. |
|  | References, 9, and 10 no page numbers while reference #24 need revision for the year. | We added the page numbers to references 9 (now reference #6) and 10 (now reference #7).  The year was corrected for reference #24 (now reference # 27). |
| Reviewer 2 | Relevant and interesting option to improve the oral health of community. There are few suggestions and 1 necessary change. | We thank the Reviewer for the positive feedback. |
|  | 1. The introduction of the abstract require some connection between 1st and 2 nd sentence. Both sentences are misfit when read in continuity. | We have now amended the introduction as suggested by the reviewer. |
|  | 2. Is the questionnaire self designed? if yes then did the authors conduct pilot study to validate it? | We conducted a pilot study, and this has been added to the method section (Page 6 lines 133-135). |
|  | 3. Title also requires change. | The title was modified to “Exploring factors influencing nurses’ attitudes towards their role in dental care” |
